# Automated testing platform for radiotherapy treatment planning scripts

**DOI:** 10.1002/acm2.13845

**Published:** 2022-11-21

**Authors:** Joseph John Lucido, Satomi Shiraishi, Srinivas Seetamsetty, David C. Ellerbusch, John A. Antolak, Douglas J. Moseley

**Affiliations:** ^1^ Department of Radiation Oncology Mayo Clinic Rochester Minnesota USA; ^2^ Department of Medical Systems Nursing, and Information Technology Mayo Clinic Rochester Minnesota USA

**Keywords:** Auotmation, Radiation Therapy, Treatment Planning

## Abstract

Realizing the potential of user‐developed automation software interacting with a treatment planning system (TPS) requires rigorous testing to ensure patient safety and data integrity. We developed an automated test platform to allow comparison of the treatment planning database before and after the execution of a write‐enabled script interacting with a commercial TPS (Eclipse, Varian Medical Systems, Palo Alto, CA) using the vendor‐provided Eclipse Scripting Application Programming Interface (ESAPI). The C#‐application known as Write‐Enable Script Testing Engine (WESTE) serializes the treatment planning objects (Patient, Structure Set, PlanSetup) accessible through ESAPI, and then compares the serialization acquired before and after the execution of the script being tested, documenting identified differences to highlight the changes made to the treatment planning data. The first two uses of WESTE demonstrated that the testing platform could acquire and analyze the data quickly (<4 s per test case) and facilitate the clinical implementation of write‐enabled scripts.

## 1 INTRODUCTION

Treatment planning for radiation therapy is a complex process, both time‐consuming and with high potential for output variation; there is great interest in streamlining and automating the process.^1^, ^2^ Automation, simplification, and standardization are recognized as highly effective means to improving patient safety,^3^ as well as reducing burden on staff. Automation can translate into lower costs for care,^2^ increased patient access,^2^ and reduced time between simulation and treatment (which can potentially improve outcomes^4^, ^5^). Automation tools for treatment planning must be adapted to meet the specific needs of the practice: many commercial treatment planning systems (TPS) include the capability to integrate user‐specific automation into the planning workflow.

Eclipse (Varian Medical Systems, Palo Alto, CA) provides an application programming interface (API) that gives mediated access to treatment planning data through user‐developed software, commonly referred to as a script. Starting in version 15, the Eclipse Scripting API (ESAPI) permits “write‐enabled” scripts to change the values of select treatment planning parameters under controlled conditions. This capability can bring significant improvements into the planning process, but the users are ultimately responsible for ensuring that the use of these scripts does not compromise patient safety or treatment quality. Software can behave in unexpected or unintended ways, due to untested assumptions or unanticipated situations; preventing these deviations from impacting users and patients demands rigorous testing during verification and validation (V&V) testing. Demonstrating the full range of situations for which the software behaves as expected requires evaluating performance on a wide range of test cases.

While testing is necessary to ensure the script is appropriate to use clinically, it is also critical to provide tools to prevent the burden of testing from becoming an obstacle to clinical implementation. We report on the development and implementation of an automated testing platform for write‐enable ESAPI scripts. We also discuss our process development framework and discuss the experience from the first two projects in our department to use this process.

## 2 METHODS

### 2.1 ESAPI overview

ESAPI manages user access to a selection of treatment planning parameters and attributes as well as the ability to execute specific commands in a similar manner to a user accessing the software through the graphical user interface (GUI) of an installed Eclipse client.^6^ The scripts can take several forms. The user can call a “plug‐in” script from within the Eclipse software: either a C#‐language text file or a compiled dynamic‐linked library (DLL). Alternatively, the script could be a compiled application that is executed independently of the Eclipse software (referred to as a “stand‐alone” application).

There are several design features of ESAPI which facilitate the safe development and use of write‐enabled scripts. First, write‐enabled scripts must first be marked as approved before they can be run in Eclipse. In general, this approval requires elevated user permissions, which are only granted to a few individuals in our department. This approval is enforced through strict version control; any change to the script requires re‐approval. In addition, the script developer must explicitly declare that the script is “write‐enabled” in the code and mark specific sections of the application to have that permission, which limits the potential to create an unintentionally write‐enabled script. Lastly, ESAPI limits the scope of access to a single patient's data at a time, limiting the potential to unexpectedly alter data for multiple patients or to overwhelm the treatment planning database with multiple concurrent requests.

### 2.2 Testing framework

Our department has instituted a software development process governance framework^7^ to manage the implementation of write‐enabled scripts. The development process is outlined in Table 1. A project review team is assigned to collaborate with the development team to develop project‐specific V&V test cases and requirements, to review the testing results, and to make a recommendation to the scripting governance group regarding the approval of the script for clinical use. The V&V testing is performed in a dedicated nonclinical development environment of Eclipse that mirrors the clinical environment. If the script is approved, final validation testing is performed in the clinical system prior to release for clinical use.

**TABLE 1:** An overview of the development process

Project stage		Involves	Description
1	Project initiated	DT	Feasibility testing and project specification
2	RT formed	GC	RT assigned, and begins discussing testing protocol
3	Development	DT	Get project ready for testing
4	Finalize testing	RT and DT	Define final testing cases, protocol, and expectations
5	Testing	DT	Perform and document specified tests
6	Review	RT	Evaluate results and make recommendation
7	Approval	GC	Determine if ready for implementation

*Note*: An overview of the development process for a write‐enabled script within the department's software development process governance framework.

Abbreviations: DT, development team; GC, governance committee; RT, review team.

### 2.3 Testing platform design requirements

The design specifications for the testing platform were

1. detect a change made by a script in any of the ESAPI‐accessible fields associated with a given patient.
2. test the scripts in a manner that is equivalent whether the end‐user will run the script as a plug‐in or stand‐alone, and whether the input is specified by command line or user input with a GUI.
3. perform tests in a reasonable amount of time.
4. straightforward specification of tests through a GUI.
5. document the results for external review.

### 2.4 V&V testing experience

For the first two write‐enabled scripting projects to use this framework, the time required to acquire and compare the snapshots was recorded, along with details about the projects and the experience of the development and review teams.

## 3 RESULTS

### 3.1 Design and implementation of testing platform

**FIGURE 1:**
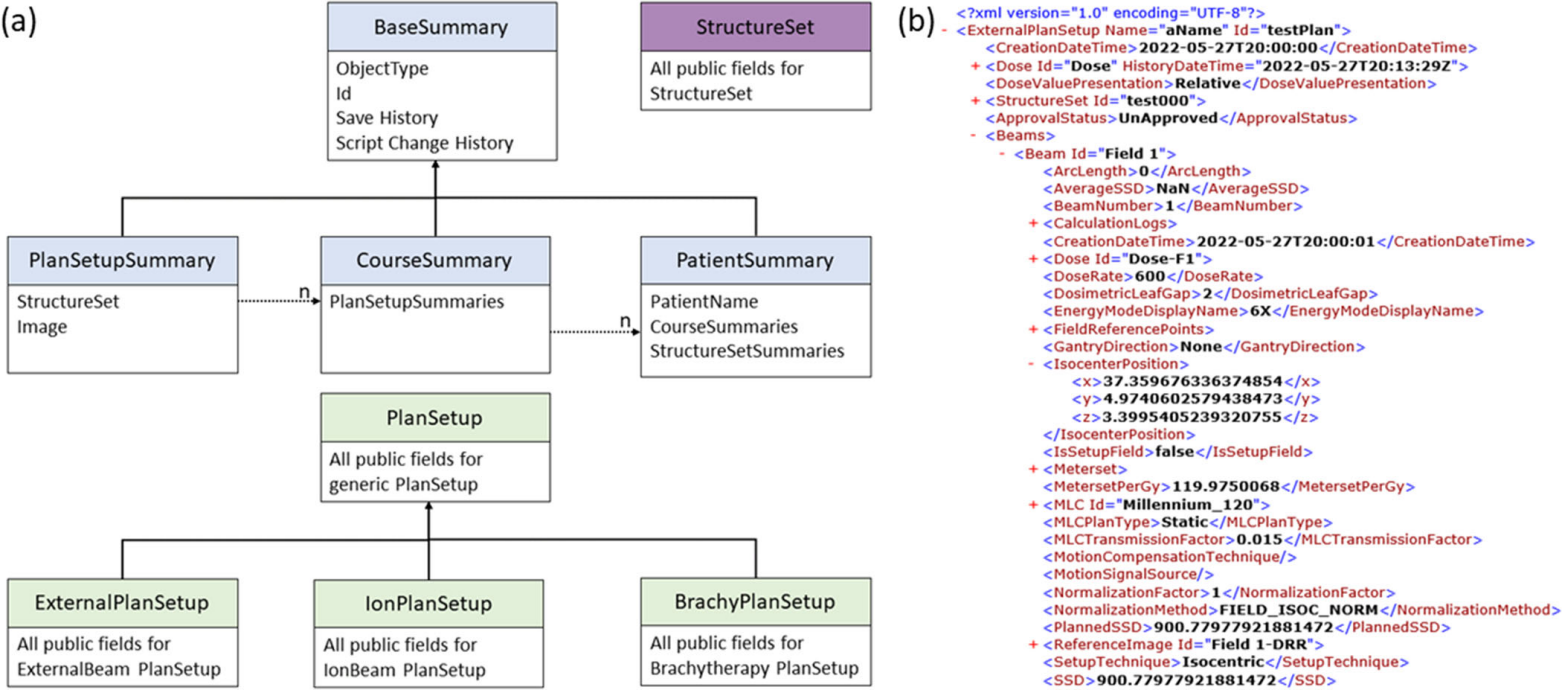
Snapshot class design. (a) Software class diagram for different types of snapshots, including high‐level summary types and detailed PlanSetup and StructureSet types. (b) Portion of a sample serialization of a ExternalBeam PlanSetup Snapshot

The testing platform was implemented as a C# application known as Write‐Enabled Script Testing Engine (WESTE). The testing process is built around the creation of a Snapshot: a file containing the data fields of the specific treatment planning data object being evaluated at a given time. Examples of data objects that can be converted to a Snapshot include a treatment plan, structure set. The Snapshot is stored as an XML file created with a custom serialization. The tester can specify one of two levels of detail for the snapshot: a detailed list of all parameters and fields associated with the object, or a high‐level summary of the object, as shown in Figure 1.

WESTE also provides the ability to identify any differences between two Snapshots (design specification #1). By comparing a Snapshot taken immediately before the execution of the script with one taken after, we can assess whether any unexpected changes occurred, as well as evaluate that the changed values were as intended. A custom module was developed to perform this task; for many treatment planning objects, we would like to compare objects based on their Id rather than the index in the list (which is assumed by off‐the‐shelf XML‐diff tools). After a comparison is run, the identified differences are presented to the user in the GUI (Figure 3), and the data can be output as an XML file for permanent documentation (further discussion of the results of this case are discussed below in Section 2.2).

**FIGURE 2:**
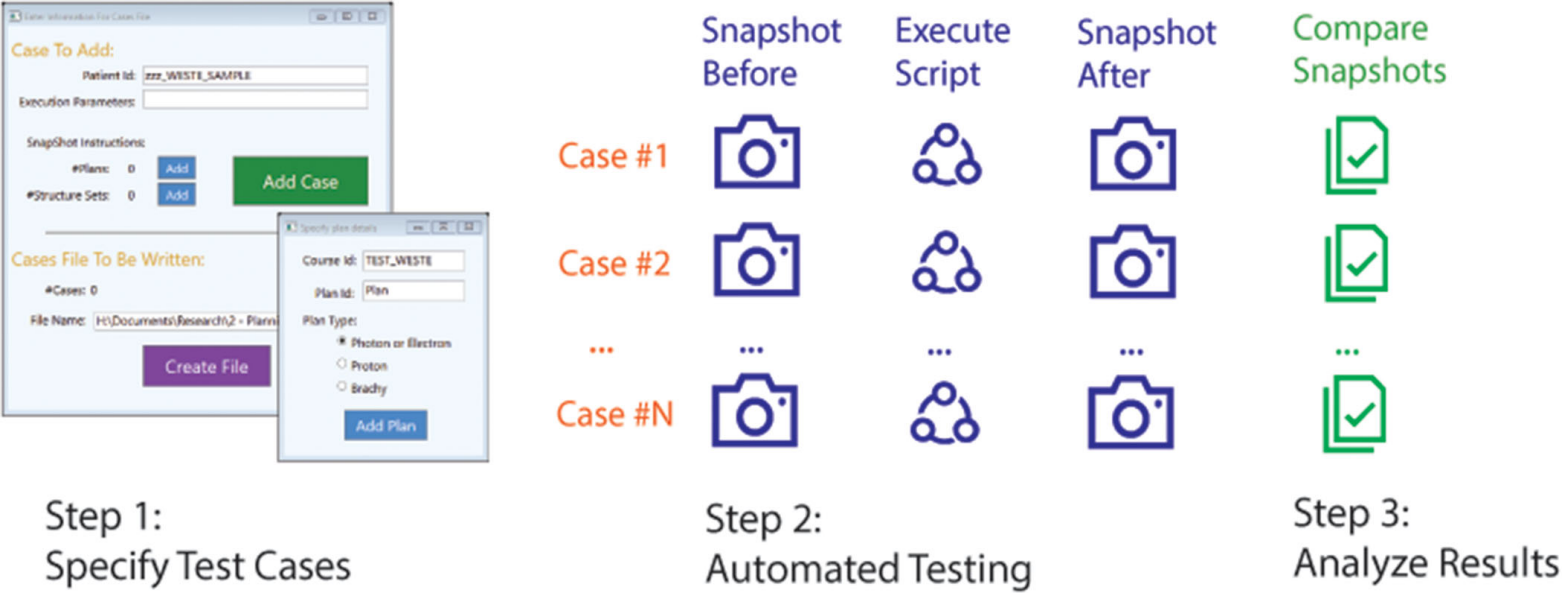
Automated Testing Process. An overview of the automated workflow for script testing with the testing platform showing the graphical user interfaces (GUI) for the test specification, a representation of the automated test execution process, and review of the comparisons

The testing workflow with WESTE is flexible to meet the needs of the tester: it can be either partially or completely automated. The user can create a Snapshots at any given time, while the comparison can be performed (and repeated) at the user's discretion. For some projects, executing the script may require substantial user input, which may mean it is most efficient to have the user execute the script manually, rather than define the input programmatically. For other situations, there could be substantial reduction in effort by performing the execution of the script and acquisition of the snapshots automatically. This workflow is represented in Figure 2. For this approach, the tester defines the cases and provides all necessary user input prior to performing the tests. WESTE acquires the initial Snapshots as specified in the input, executes the script from the command line as a stand‐alone executable for each case, and takes the final Snapshots. Once the tests are completed, the comparisons are displayed in the GUI.

For the V&V testing to be valid, the test must accurately reflect the behavior of the script under clinical conditions (design specification #2). The automated workflow requires running the script as a stand‐alone executable, with input specified programmatically (usually with an input file), while in clinical use the script takes in input from a GUI and may be executed as plug‐in script. To bridge this gap, a script template was developed as shown below in Figure 4. One key feature of this template is that it automatically detects if it has been executed as a plug‐in or stand‐alone and then acquires the input using the appropriate means – either an interactive GUI for the user, or an input file specified by the testing platform. Either way, this input information is processed into a common data object (the “Input Object” in Figure 4) and passed to the write‐enabled portion of the project (“Test Application”), and likewise the template directs the output data appropriately. This decouples the input and output sections of the code from the “write‐enabled” portion, which helps streamline the testing process. In addition, the template provides a wrapper that recreates the way Eclipse interacts with a plug‐in script, so that the automated workflow can be used for plug‐ins. Once testing is completed, only the script file or DLL itself needs to be moved to the script directory and get approved.

After the initial serialization of the Snapshots, the testing process defined here does not require interfacing with the treatment planning database, which allows for efficient data‐processing. The time required to acquire a single snapshot varies depending on the complexity of the data for the patient. However, for the projects discussed below, the snapshots never required more than 3 s to acquire or analyze. The user can input the test specifications through the GUI as shown in Figure 2, and the input is stored as a text file to facilitate repeat testing. In addition, an API has been developed for WESTE to allow the input files to be generated programmatically, which can streamline the workflow further for complex testing regimes. In the end, the use of WESTE represents a small fraction of the total development time, and the user experience has been designed to minimize the learning curve for testers (design specification #3). The test results are documented as an XML file (as are the Snapshots), which the user can retain to demonstrate that the testing requirements have been satisfactorily completed, and as a baseline for future regression testing (design specification #4).

WESTE was validated and commissioned prior to being released for evaluation of clinical scripts. A series of test cases were established by comparing Snapshots taken before and after known changes were introduced, to ensure that the expected changes were identified by WESTE (as well as no unexpected changes). These test cases were chosen to reflect a wide variety of realistic and unrealistic clinical scenarios, with many different levels of complexity and covering multiple conditions (such as approval status, multiple courses). This also included situations in which no changes were made, to ensure that no false positives were detected. The lead designer also used their routine clinical work to generate tests for WESTE, to ensure that the test cases represented realistic scenarios. Finally, all tests cases were independently verified by hand for the first project that utilized WESTE. For subsequent projects, a subset of cases was always verified by hand, in addition to all unexpected findings detected by WESTE. This testing will need to be repeated in response to any update or new version of either the TPS or WESTE.

### 3.2 Experience with WESTE

#### 3.2.1 Project #1: Automated planning

The first write‐enabled project to use WESTE for V&V testing was a stand‐alone executable that creates a parallel‐opposed megavoltage X‐ray treatment plan on a diagnostic computed tomography (CT) image or a cone‐beam CT acquired on a treatment linear accelerator. The primary purpose of this application is to streamline the planning process for emergent patients (particularly outside of regular treatment hours) without resorting to the treatment unit's unplanned treatment mode functionality or a formal CT simulation and treatment plan creation by a dosimetrist. This application requires a large amount of direct user input, and the V&V testing also served as an opportunity to get end‐user feedback on the script functionality, so the semi‐automated testing workflow was used. In the development environment, the testing protocol specified 20‐specific test cases. After completion of the planning process and treatment delivery to a phantom, initial chart checks (similar to the plan review by a physicist prior to first treatment) were performed independently by two experienced physicists, in addition to a comparison of the Snapshots by WESTE. Since the write‐enabled script was intended to create an entirely new treatment plan, the primary role of the snapshot comparison was to verify and document that no other courses, structure sets, or plans were modified by the script, while the results were also useful to the chart checking physicists because they presented the plan parameters in an organized manner. No issues of concern were identified during the final set of tests by the testers or a review of the Snapshots. As shown in Table 2, the execution time for WESTE during this project was very short. Timing data were not recorded for the time required for the manual review of the data during the initial chart checks. However, subsequent clinical experience with the planning script indicates that this process generally takes 5–10 min. Since the WESTE documentation presents the data in an organized and streamlined manner, rather than requiring the user to navigate through various windows and workspaces in the TPS, we could plausibly estimate that WESTE would save 10% of the total time in this context, or about 0.5 min per individual chart check (or 1 min per case), and 20 min overall.

**TABLE 2:** Timing results for test platform performance for evaluation projects

			Total execution time for WESTE					
Evaluation project		#Test cases	Initial snapshot	Final snapshot	Comparison	Mean execution time for WESTE per case	Estimated review time per case	Estimated time‐saving per case
#1	Parallel‐opposed plan creation	20	18 s	22 s	25 s	3.3 s	300 s	120 s
#2	Plan nomenclature	16	12 s*	11 s*	6 s*	2.4 s*	60 s	60 s

*Note*: Timing results for the use of the automated test platform for two write‐enabled script projects, including the number of cases run as part of the test protocol, the execution time for WESTE, and the estimated time for manual review of the data and time‐savings estimated due to the use of WESTE. (*)Times were not recorded for the initial test, these measurements were subsequent repeat test with equivalent cases.

#### 3.2.2 Project #2: Plan labeling

A write‐enabled script was created to assist the planning team in labeling a treatment plan and associated course in accordance with the department's standard nomenclature. This script prompts the user to specify the necessary information about the patient's treatment with a GUI, which is then used to suggest the appropriate Id for the treatment course and plan. The user can only select values from pre‐specified lists, which places strict boundaries on the way that the user can interact with the script. If the plan is unapproved, the script will automatically assign those Ids. Due to the simplicity of the user interaction and small scope of the testing protocol, the developer opted to use the semi‐automated workflow. The testing protocol specified 16 test cases to run on the development Eclipse system; the cases covered a variety of disease sites, plan approval statuses, and number of treatment plans and courses for the given patients. The time required for the execution of WESTE was not recorded for this project; a repeat test performed using a similar set of patient data was performed and demonstrated that execution was quick (as shown in Table 2). As this was an early clinical use of WESTE, all data were manually verified as well, and it was estimated that this took around 2 min per case, or about 30 min total.

**FIGURE 3:**
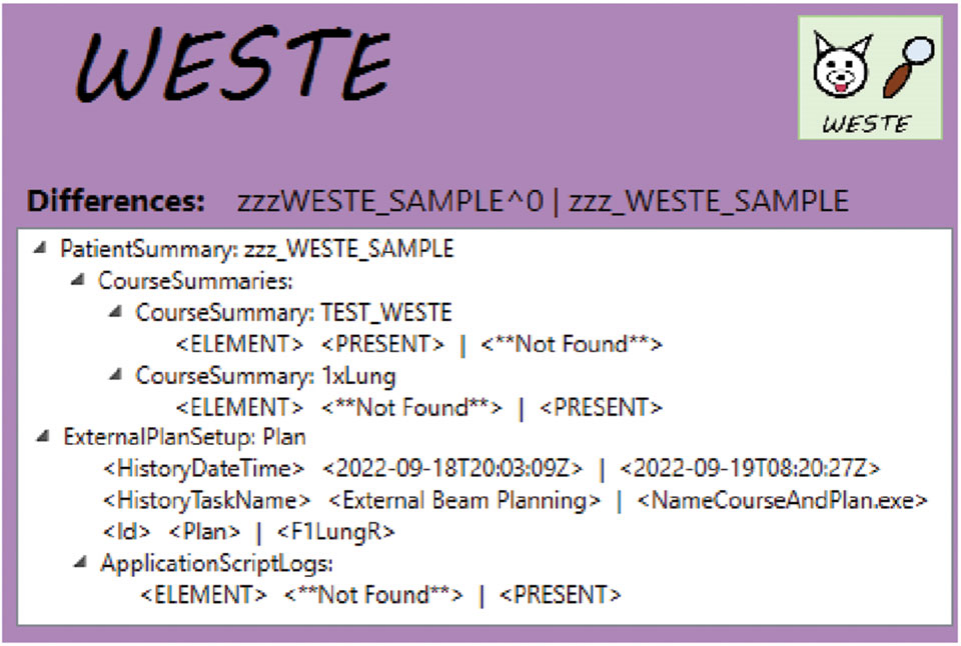
Review of the database change after execution of script. Review of the changes detected in the database after the execution of the plan labeling script (see Section 2.2), showing both the patient summary comparison and the plan. These records show that both the plan and the course Ids were changed, as expected. In addition, the plan history and ApplicationScriptLog shows that a change was made by the executable NameCourseAndPlan.exe.

A comparison window showing the differences detected before and after the running the application (called NameCourseAndPlan.exe) for a sample case is shown in Figure 3 . For this example, there was initially a course labeled “TEST_WESTE” containing a single external beam plan (“Plan”). The script was run to change the labels of both the course and plan to confirm with the departmental nomenclature, so they were changed to “1xLung” and “F1LungR”, respectively. The first six lines of the result section refer to the snapshot for the Patient Summary, indicating that WESTE correctly identified that the course changed name as expected during the execution of the script. The next section compares the plan, showing that the Id was changed as intended. It also shows the expected changes to the save history of the object and the addition of an ApplicationScriptLog indicating that changes were made by the NameCourseAndPlan script. This analysis shows that the script made the intended changes without introducing any unexpected ones, confirming the script execution was successful.

**FIGURE 4:**
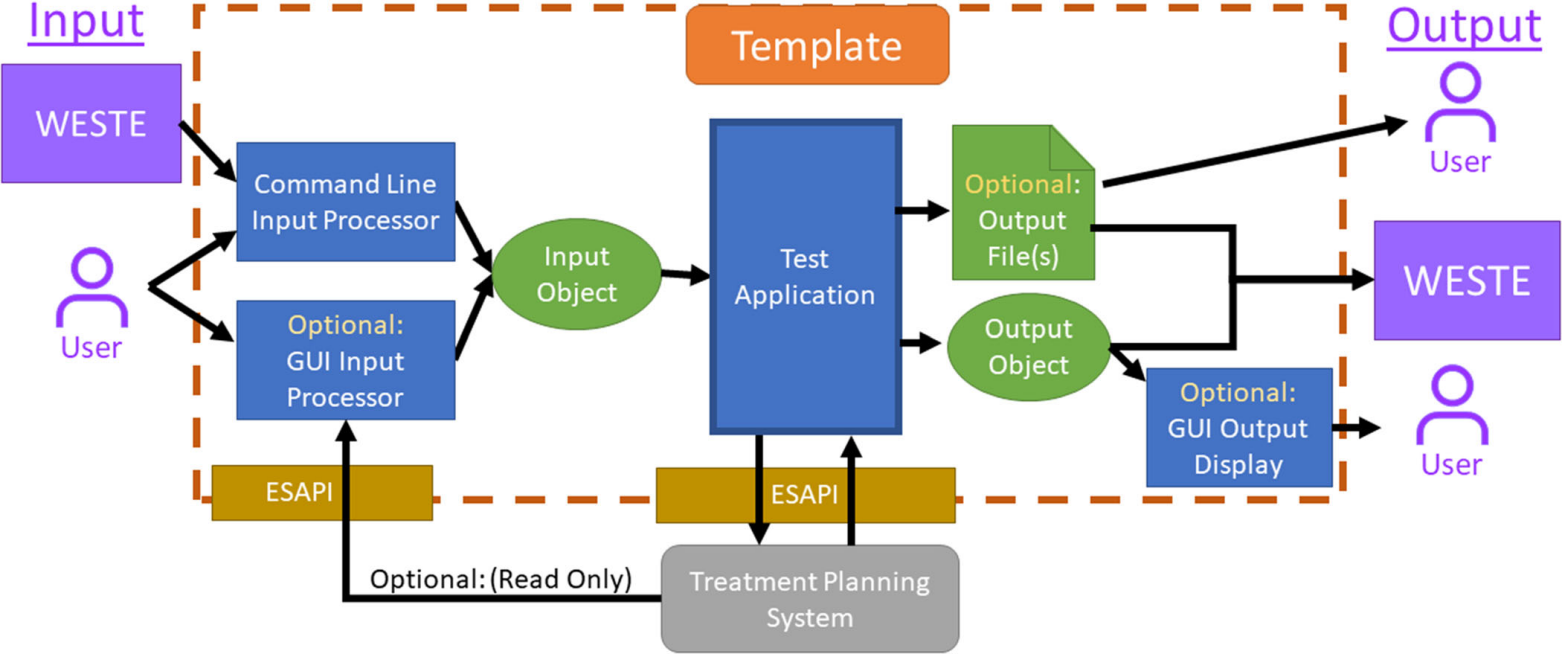
Script template for automated testing. Software design of the script template enabling automated testing

## 4 DISCUSSION

WESTE successfully met the design specifications. The software has proven easy to use for multiple developers, and the execution time is insubstantial compared to the other aspects of testing and development. Importantly, the testing platform provides transparent documentation that sufficient V&V testing has been performed and facilitates on‐going regression testing of the scripts. WESTE makes it straightforward for the testing and development teams to identify the changes that have been made in the database by the script. This makes it very effective at bringing unintended changes to the tester's attention. However, since the motivation for using write‐enabled scripts is to make changes to the data, and many of the most valuable applications of write‐enabled scripting involve changing a large number of parameters or the creation of entirely new planning objects such as structure sets or treatment plans (for example, project #1 discussed above). In these situations, simply comparing the changes to the database is insufficient to assess the correctness of those changes—an experienced user will need to thoroughly review them, and additional quality assurance (QA) may also be warranted (such as an independent MU calculation and/or delivery QA). This can demand a high‐level of familiarity with the treatment planning process and the database, as well as considerable time. WESTE can also be used to facilitate this review by enabling the user to compare the post‐execution snapshot with a snapshot for a manually created “gold standard” that represents the intended final state of the data. In addition to showing the potential to streamline the testing procedure, most importantly it added value to the process by ensuring that all parameters were checked, and that the data were presented in a way that it made it easy for the user to verify the correctness of the changes.

There have been challenges associated with implementation of the testing platform. A radiation oncology information system's primary purpose to ensure data integrity, which is incompatible with the ability to revert the database to an initial state that is desirable in a testing environment. Since we require that the test environment mirrors the clinical system, the burden of data management falls on the testers and developers. To streamline the testing process, we suggest that the vendor could add this type of data management as a part of development and testing oriented systems. Furthermore, it is important to instill a “test‐first” culture among developers. The primary goal of the project should be to pass a battery of tests successfully as much as fulfill a clinical function. If developers perceive that the tests are not an accurate representation of the clinical use, that is a shortcoming of the selected testing protocol rather than the methodology.

Fortunately, no unexpected behavior has been detected by WESTE. This reflects the skill of the developers as well as the rigorous testing performed prior to submitting the project for formal V&V testing, but it does limit the study in terms of providing quantitative data about the detectability of errors. During the development of WESTE, myriad changes were intentionally introduced to test their detectability, but since these were known a priori, they do not provide data about real‐world performance.

WESTE provides an automated test platform for V&V testing of user‐developed scripts. This assures that the scripts are safe to use while reducing the testing burden on the development team. Incorporating WESTE into a software development framework has enabled the introduction of write‐enabled scripting into clinical practice.

## AUTHOR CONTRIBUTIONS

All authors were participants in the Scripting Governance Group, which created the specifications for the script testing platform and discussed and approved the implementation, as well as reviewed the results of the two example projects discussed. JJL and SS were responsible for developing the two example projects. JJL developed the scripting platform. All authors were involved in the writing and review of the manuscript.

## CONFLICT OF INTEREST

The authors declare that there is no conflict of interest that could be perceived as prejudicing the impartiality of the research reported.
